# Imaging features of granular cell tumor in the breast: Case report

**DOI:** 10.1097/MD.0000000000023264

**Published:** 2020-11-20

**Authors:** Qiao Zeng, Lan Liu, Qingyi Wen, Liping Hu, Linhua Zhong, Yongjie Zhou

**Affiliations:** Department of Radiology, Jiangxi Cancer Hospital, Nanchang, Jiangxi, China.

**Keywords:** breast carcinoma, breast mass, breast pathology, granular cell tumor, magnetic resonance imaging

## Abstract

**Rationale:**

Granular cell tumor of the breast (GCTB) is a benign rare tumor. There are limited reports on its imaging manifestations. GCTB is often misdiagnosed as breast cancer, which results in unnecessary radical mastectomy and excessive treatment. In this article, we have reported a case of a 56-year-old postmenopausal woman with GCTB and highlighted the imaging features to differentiate this rare tumor from breast cancer.

**Patient concerns:**

A 56-year-old postmenopausal patient had a chief complaint of a subcutaneous nodule in the upper outer quadrant of her right breast for 2 months. She underwent physical examination, color Doppler ultrasonography, mammography, magnetic resonance, and postoperative pathology.

**Diagnoses:**

The final diagnosis was GCTB. The tumor cells were intermingled with the fibrous stroma and normal breast parenchyma and showed positive immunoreaction to S-100, CD68, and neuron-specific enolase.

**Interventions:**

The patient underwent lumpectomy and sentinel lymph node biopsy.

**Outcomes:**

The patient recovered well after lumpectomy and had no complications during the 2-year follow-up.

**Lessons:**

There are some important imaging features of GCTB that can be used to distinguish it from breast carcinoma to reduce misdiagnosis.

## 1 Introduction

Granular cell tumor (GCT) is a rare, benign neoplasm with a myogenic origin that was first identified in the tongue in 1854 by Weber and was then described in the breast and tongue by Abrikossoff.^[1]^ It is assumed to originate from perineural or putative Schwann cells of the peripheral nerves or their precursors that grow in the lobular breast tissue owing to its immunohistochemical features, such as strong S-100 positivity.^[2]^ GCT is rare in the breast and frequently mimics carcinoma in clinical, radiological, and frozen section examinations. Misdiagnosis of this tumor can lead to radical mastectomy and may result in unnecessary therapy.^[3]^ The objective of the present case report was to document a case of GCT of the breast (GCTB) in a postmenopausal woman and to highlight the imaging features in the differential diagnosis of this rare tumor to reduce misdiagnose.

## 2 Case presentation

**Figure 1:**
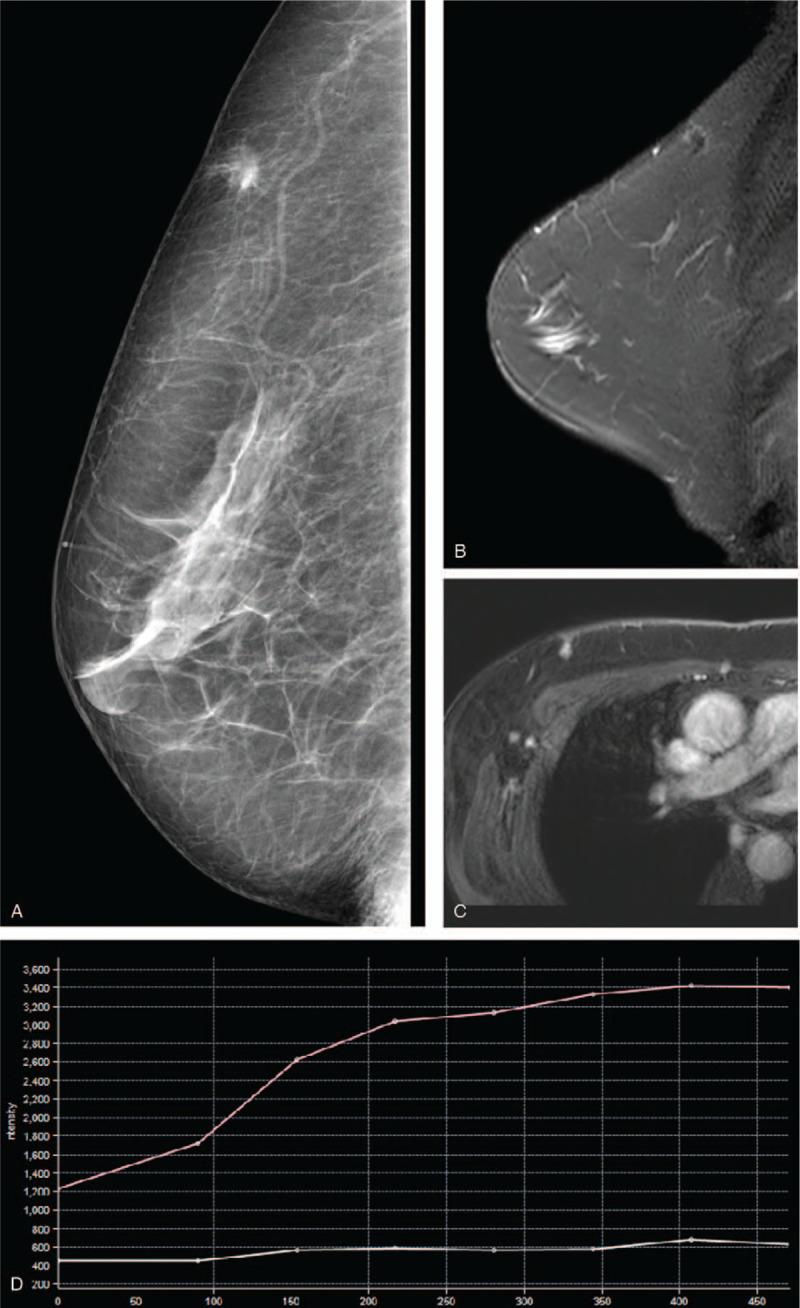
(A) Mediolateral oblique mammogram shows a subcutaneous mass with spiculate boundary and adhesion to the adjacent skin. Magnetic resonance imaging shows an irregular nodule with an isointense signal on T2-weighted sequence image (B), uniformly enhanced after gadolinium injection (C) and I type of TIC curve (D). TIC = time-signal intensity curve.

**Figure 2:**
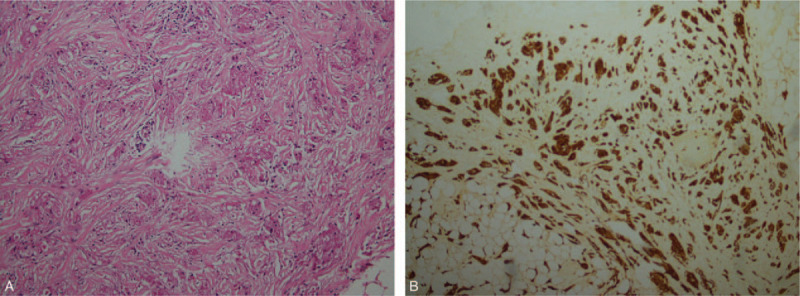
(A) Microscopy imaging shows that tumour cells are polygonal, rich in cytoplasm, filled with eosinophilic particles, and infiltrating into the surrounding (HE × 200). (B) Immunohistochemistry shows diffuse reactivity for S100 protein.

A 56-year-old postmenopausal woman inadvertently found a subcutaneous tumor in the right breast before 2 months and was admitted to our hospital. Written informed consent was provided by the patient for this case report. Physical examination revealed a nodule at 10 o’clock in the upper outer quadrant of the right breast, which was approximately 1 cm in diameter, was hard on palpation, had undefined boundaries, has poor mobility, and had no tenderness. Axillary lymphadenopathy was not observed. Laboratory tests revealed normal findings. Ultrasonography showed the hypoechoic mass was approximately 0.8 × 0.9 cm in size and had blurry borders, an irregular shape, and attenuated rear echo. In addition, color Doppler flow imaging showed that the edge of the mass had a slight blood flow signal. Mammography confirmed the presence of irregular nodules with spiculate boundaries, higher density than normal glands, and adhesion to the adjacent skin without calcification (Figure 1A). Magnetic resonance imaging (MRI) of the breast revealed that the lesion had hypointense signal on T1-weighted imaging and isointense signal on T2-weighted and diffusion-weighted imaging compared with the adjacent glandular tissue. On dynamic enhancement, the mass was progressively enhanced, and the time-signal intensity curve (TIC) shape was I style (Figure 1B-D). According to its radiology manifestations, a Breast Imaging Reporting and Data System Category 4C was provided by radiologists. The patient was advised to undergo lumpectomy and sentinel lymph node biopsy. Postoperative pathology reported GCTB with positive immunoreaction to S-100, neuron-specific enolase, and epidermal growth factor receptor and negative immunoreactivity to cytokeratin, oestrogen, and progesterone, and HER2/Neu receptors (Figure 2A-B). The patient recovered well after surgery and was followed up for 2 years. The study was approved by the Institute Research Ethics Committee of Jiangxi Cancer Hospital and the informed consent was given by the patient.

## 3 Discussion

The incidence of GCTB is very low, accounting for 5% to 8% of GCT and 0.1% of breast tumors; it usually occurs in premenopausal African-American women aged 30 to 50 years.^[4]^ The youngest case of GCTB was pertained to 9-year-old patient.^[5]^ GCTB is usually benign, although 1% cases can be malignant and 10% cases can be associated with breast cancer. Most cases of GCTB are incidentally found in painless masses, especially in the subcutaneous area, that have a hard texture, an unclear border, slow growth and are adhered to the skin.^[6]^ GCTB generally shows benign biological behaviour and malignant clinical signs. When tumors present malignant features, such as size >5 cm, rapid growth, adjacent structure invasion, and axillary lymphadenopathy, it suggests the possibility of malignant GCTB.^[7]^ Clinically and radiographically, GCTB is easily misdiagnosed as breast cancer owing to its low incidence and lack of specificity, which results in unnecessary radical mastectomy and excessive treatment.^[3]^ Typically, GCT presents abundant granular eosinophilic cytoplasm on microscopy, from which this tumor derives its name. The antibodies used to confirm the diagnosis of GCT by immunohistochemistry are S100 and CD68.^[8]^ According to the recommendations of the European Breast Association, histopathological examination using the core biopsy is the gold standard for the diagnosis of GCTB, while fine-needle aspiration is often illegible owing to its unclear results.^[9]^ Local enlargement resection with negative margins is the main treatment for benign GCTB, and the prognosis is excellent.^[6]^

Imaging features of GCTB is not specific and often overlaps with breast cancer. Ultrasound, mammography, and MRI of GCTB are often diagnosed as Breast Imaging Reporting and Data System Category 4-5. Ultrasonography, the common features of GCTB are irregular star-shaped hypoechoic masses with unclear boundaries and attenuation of the rear echo. Color Doppler flow imaging cannot probe within the blood flow signal in the tumor owing to the attenuation of the inner and rear echoes.^[11]^ Mammographically, GTCB presents as a solid mass with blurry edges and rarely calcifications.^[12]^ The tumor generally shows isointense or slightly hyperintense on T2-weighted sequence images, which is considered a typical aspect of GCTB in MRI studies.^[13]^ Enhancement of GCTB is variable, ranging from ring-like to intense homogeneity. The TIC shape of this tumor is mostly type I and II.^[14]^ In addition, GCTB does not show increased glucose metabolism on fluorodeoxyglucose positron emission tomography (FDG-PET). Therefore, FDG-PET can provide more information for differentiating GCTB from malignant tumors.^[15]^

It is difficult to distinguish GCTB from breast cancer for radiologists, and the diagnosis mainly depends on histopathology and immunohistochemistry. The imaging manifestations of GCTB are a consequence of the histopathological characteristics of the tumor; therefore, GCTB may be distinguished from breast cancer by the following aspects. First, GCTB is likely to be derived from Schwann cells of the supraclavicular nerve and has infiltrative growth, resulting in an irregular subcutaneous tumor with blurry edges on images, but there is no oedema in the rims of the lesion compared with breast cancer.^[10]^ Second, the homogeneity of GCTB tumor cells leads to a uniform echo/density/signal, unlike the heterogeneity and microcalcification of breast cancer. Third, T2-weighted imaging and diffusion-weighted imaging signals in GCTB are often lower than those in breast carcinoma, while the TIC shape of GCTB is mainly I or II type compared with III type for breast cancer. Lastly, GCTB generally manifests benign biological behaviour during follow-up, while breast cancer is associated with rapid growth, adjacent structure invasion, and axillary lymphadenopathy.

In conclusion, there are important imaging features of GCTB. Simultaneously, by comprehending the histopathological characteristics, being acquainted with imaging manifestations, and associating the findings with benign biological behaviour, it is possible for radiologists to distinguish GCTB from breast carcinoma.

## Acknowledgments

We thank the patient and contributing doctors for participation in this study. Meanwhile, we would like to thank Editage (www.editage.cn) for English language editing.

## Author contributions

**Conceptualization:** Qiao Zeng, Lan Liu.

**Data curation:** Qiao Zeng, Qinyi Wen, Liping Hu, Linhua Zhong.

**Supervision:** Lan Liu, Yongjie Zhou.

**Validation:** Lan Liu.

**Visualization:** Linhua Zhong, Yongjie Zhou.

**Writing – original draft:** Qinyi Wen, Liping Hu.
